# Recovery of Viral RNA and Infectious Foot-and-Mouth Disease Virus from Positive Lateral-Flow Devices

**DOI:** 10.1371/journal.pone.0109322

**Published:** 2014-10-14

**Authors:** Veronica L. Fowler, Bartlomiej M. Bankowski, Bryony Armson, Antonello Di Nardo, Begoña Valdazo-Gonzalez, Scott M. Reid, Paul V. Barnett, Jemma Wadsworth, Nigel P. Ferris, Valérie Mioulet, Donald P. King

**Affiliations:** 1 Vesicular Disease Reference Laboratory, The Pirbright Institute, Pirbright, Surrey, United Kingdom; 2 Animal Health and Veterinary Laboratories Agency (AHVLA), New Haw, Surrey, United Kingdom; 3 Veterinary Medicines Directorate, New Haw, Surrey, United Kingdom; 4 Institute of Biodiversity, Animal Health and Comparative Medicine, College of Medical, Veterinary and Life Science, University of Glasgow, Glasgow, United Kingdom; Centers for Disease Control and Prevention, United States of America

## Abstract

Foot-and-mouth disease Virus (FMDV) is an economically important, highly contagious *picornavirus* that affects both wild and domesticated cloven hooved animals. In developing countries, the effective laboratory diagnosis of foot-and-mouth disease (FMD) is often hindered by inadequate sample preservation due to difficulties in the transportation and storage of clinical material. These factors can compromise the ability to detect and characterise FMD virus in countries where the disease is endemic. Furthermore, the high cost of sending infectious virus material and the biosecurity risk it presents emphasises the need for a thermo-stable, non-infectious mode of transporting diagnostic samples. This paper investigates the potential of using FMDV lateral-flow devices (LFDs) for dry transportation of clinical samples for subsequent nucleic acid amplification, sequencing and recovery of infectious virus by electroporation. FMDV positive samples (epithelial suspensions and cell culture isolates) representing four FMDV serotypes were applied to antigen LFDs: after which it was possible to recover viral RNA that could be detected using real-time RT-PCR. Using this nucleic acid, it was also possible to recover VP1 sequences and also successfully utilise protocols for amplification of complete FMD virus genomes. It was not possible to recover infectious FMDV directly from the LFDs, however following electroporation into BHK-21 cells and subsequent cell passage, infectious virus could be recovered. Therefore, these results support the use of the antigen LFD for the dry, non-hazardous transportation of samples from FMD endemic countries to international reference laboratories.

## Introduction

Foot-and-mouth disease (FMD) virus (FMDV) (family *Picornaviridae*, genus *Aphthovirus*) [1] causes a highly infectious and contagious disease of wild and domesticated cloven-hoofed animals, with huge potential for rapid spread within susceptible animal populations. There are seven immunologically distinct serotypes with more than sixty antigenic variants of the virus. FMD is endemic in large parts of Asia, Africa and some parts of South America [2]–[3]. Based on samples received to the Office International des Epizooties (OIE)/Food and Agriculture Organisation of the United Nations (FAO) FMD laboratory Network, FMD viruses of type O are the most frequently detected serotype, followed by serotype A. Serotype C has not been isolated from field cases since 2004, while the southern African serotypes (SAT) 1 to 3 are usually, but not exclusively, found in sub-Saharan Africa [4].

In developing countries, accurate and timely diagnosis and reporting of FMD outbreaks is often hindered by low levels local of infrastructure, such as lack of reliable power supply, inadequate numbers of suitably trained personnel as well as lack of resources within national and regional reference laboratories. Therefore many countries rely on the services of international laboratories such as those within the OIE/FAO FMD Laboratory Network to confirm and characterise the strain of virus collected in clinical material from suspected outbreaks. However, transportation of potentially infectious samples from the field to these international centres, poses significant costs and logistical challenges. In particular, maintenance of the cold chain is important since inadequately preserved samples are often degraded which generates difficulties for the reporting laboratory to fully characterise the field isolate, resulting in a loss of vital information required for control measures to be implemented. In addition, sending live infectious FMDV represents a major biosecurity risk, in that it could cause further outbreaks in the country of origin, or result in a major disease incursion elsewhere, including a disease free country, should the sample fail to be handled correctly during transport. As a result, there is interest in developing and evaluating methods that can be used to preserve clinical samples at source in a form that is non-infectious and less subject to degradation during transit, but which can be fully recovered upon arrival at the reference laboratory.

There is a growing body of evidence demonstrating that preservation of clinical samples at source and full recovery within a laboratory is indeed possible. Hofmann et al, [5] reported that FMDV and Classical Swine Fever Virus (CSFV) could be recovered via electroporation from samples stored in Trizol, whilst more recently Belsham et al, [6] and Bisht et al, [7] demonstrated that live FMDV can also be recovered from commercially available RNA storage buffers via electroporation [6] and transfection [7]. Both methods, however, still require the transport of liquids which is not ideal due to the need for appropriate high bio-secure packaging, maintenance of the cold chain and also the potential risk of leakage and thus loss of sample material. In this study we investigated whether a FMDV antigen lateral flow device (LFDs) (SVANODIP FMDV-Ag) could be used for dry transportation of FMDV for later nucleic acid amplification, sequencing and recovery of live virus.

## Materials and Methods

### Viruses

Representative archival material from the FAO World Reference Laboratory for FMD (WRLFMD), Pirbright, UK was used in this study. These were isolates from cell culture (CC) and epithelial suspensions (ES) of clinical samples from four FMDV serotypes A, O, Asia 1 and SAT 1 (Table 1).

**Table 1:** Foot-and-mouth disease virus samples used in the study.

Virus	Sample type	Serotype
TUR 8/1969	ES	FMDV O
HKN 10/2005	CC	FMDV O
IRN 53/2006	CC	FMDV O
UKG 7B/2007	ES	FMDV O
BAR 2/2008	CC	FMDV O
KUW 2/2008	CC	FMDV O
SAU 3/2008	CC	FMDV O
O_1_BFS Field strain	CC	FMDV O
O_1_BFS Cell culture adapted virus	CC	FMDV O
TUR 20/2006	CC	FMDV A
IRN 36/2007	CC	FMDV A
IRN 1/2008	CC	FMDV A
KEN 8/2008	CC	FMDV A
BAR 4/2009	CC	FMDV A
TUR 4/2013	ES	FMDV A
ZAM 5/2008	CC	FMDV SAT 1
IRN 15/2001	CC	FMDV Asia 1
PAK 9/2013	ES	FMDV Asia 1
TUR 2/2014	ES	FMDV Asia 1

ES: Tongue epithelial suspension. CC: Cell culture supernatant.

### Synthetic FMDV RNA

Synthetic viral RNA was generated from a plasmid pT73S containing full-length genomic sequences of FMDV serotype O [8] by *in vitro* transcription using a commercially available T7 RNA polymerase kit (MEGAscript T7 Transcription Kit, Life technologies, UK) following manufacturer's guidelines. This RNA was then purified using a MEGAclear kit following manufacturer's guidelines (Life technologies, UK) prior to being used as a positive FMDV RNA control within the electroporation studies.

### Epithelial suspension preparation and testing on Lateral Flow Devices (LFDs)

ES were prepared using the SVANODIP FMDV-Ag Extraction kit (Svanova, Uppsala, Sweden) following manufacturer's guidelines. ES were added to FMDV antigen LFDs (SVANODIP FMDV-Ag LFD, Svanova Biotech AB) as previously published [9] and following manufacturer's guidelines. Where CC derived viruses were used, 6 drops of the cell culture supernatant were added to each LFD. All LFDs were incubated for 10 minutes at room temperature (RT) to allow a positive result to develop at the test-line. For time course evaluation studies, LFDs were sealed in 50 ml falcon tubes or petri dishes with a desiccant and stored at RT or 37°C.

### Elution of nucleic acid from LFDs

**Figure 1:**
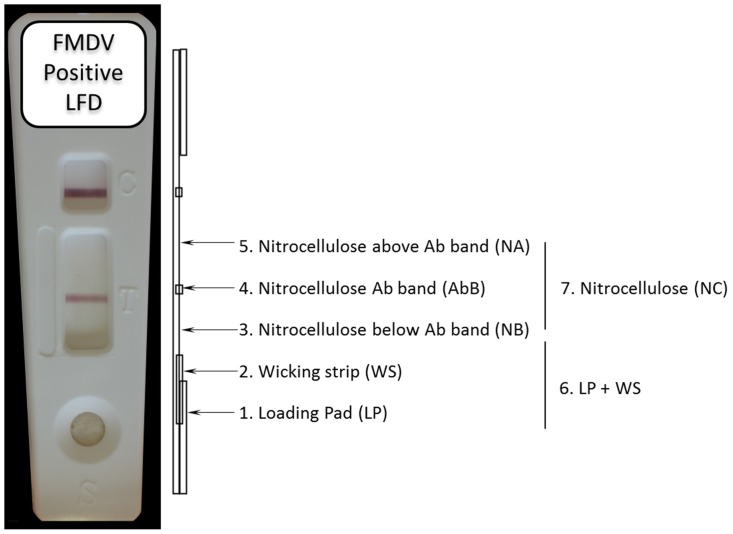
Illustration of a representative FMDV positive LFD and lateral view sketch of internal sections that were used to evaluate the recovery of RNA; LP: Loading Pad; WS: Wicking strip; NB: Nitrocellulose below Ab Band (NB); Nitrocellulose Ab Band (AbB); Nitrocellulose above Ab band (NA).

Nucleic acid was eluted from five individual sections (1–5; loading pad (LP): wicking strip (WS): nitrocellulose below antibody band (NB): nitrocellulose antibody band (AbB): nitrocellulose above antibody band (NA) or two combined sections (6–7; LP+WS; NC) of the FMDV positive LFDs (Figure 1). These sections were excised from each LFD using clean, sterile forceps and scalpel blade for each device and individually mixed with 100 µl of elution buffer consisting of RNase inhibitor (20 mM HEPES-KOH, pH 7.6; 50 mM KCl; 8 mM DTT; and 50% (v/v) glycerol - 20 Units/µL, Applied Biosystems, UK) and sterile nuclease-free water (Ambion, UK) at 1∶50 dilution. Where sections were combined (6–7; LP+WS; NC) these were also added to a total of 100 µl of elution buffer. These sections were removed from the LFD and added directly to the elution buffer and incubated at RT for 5 minutes. These tubes were then centrifuged at 3489*xg* (Hettich Rotanta 460R) for two minutes to obtain the eluted nucleic acid. In the case of nitrocellulose (NC) sections, the LFD was scraped from its plastic backing using a scalpel blade with the scrapings added directly into 100 µl of elution buffer. Eluted nucleic acid preparations were stored at −80°C until use.

### Real-time rRT-PCR on FMDV RNA eluted from the LFDs

To assess the quality of nucleic acid that was recovered from the LFDs, reverse transcription real-time PCR (rRT-PCR), utilising primers and TaqMan probes targeting the 3D region (RNA polymerase) of the FMDV genome [10], was performed according to the protocol as previously described [11]. To determine the limit of detection of the elution process, parallel decimal dilution series (1∶10, 1∶100, 1∶1K, 1∶ 10K, 1∶100K) of ES from virus isolate BAR 2/2008 was prepared and applied to LFDs. Eluted nucleic acid from LP, WS and AbB was subsequently tested by the 3D rRT-PCR as described above.

### Real-time rRT-PCR on FMDV RNA extracted directly from ES

The ability to detect FMDV-specific RNA by 3D rRT-PCR in the elution wash from the positive LFDs was directly compared to RNA extracted from the original sample that had been added to the LFD. In these experiments, RNA was prepared with an automated programme (MagNa Pure LC Robot, Roche, UK) routinely used for extraction of FMDV RNA from clinical samples [12].

### Amplification and sequencing of VP1 from FMDV eluted from the LFD

To determine whether VP1 sequences could be generated using RNAs recovered from the LFD, a one-step RT-PCR was performed [13] utilising forward primers O-1C244F (5'-GCAGCAAAACACATGTCAAACACCTT-3') or O-1C272F (5'-TBGCRGGNCTYGCCCAGTACTAC-3') and reverse primer EUR 2B-52R (5'-GACATGTCCTCCTGCATCTGGTTGAT-3') on elution wash collected from LFDs which had been stored at RT for one month. After PCR, amplification products were analysed by electrophoresis on a 1.5% agarose gel. Fragments of appropriate size (O-1244F/EUR2B-52R – 1162-1165 and O-1272F/EUR2B-52R – 1132-1135 nt) recovered from the gel were purified (Illustra GFX PCR DNA and Gel Band Purification Kit, GE Healthcare Life Sciences, UK) as per the manufacturer's instructions prior to sequencing as previously described [13]. The raw data were assembled using the Lasergene 11 suite (DNASTAR, Madison, WI) and further sequence analysis performed using BioEdit (version 7.0.1) [14].

### Amplification of the complete FMD viral genome from RNA eluted from the LFD

To determine whether sequences comprising the complete FMD viral genome were recoverable from LFDs, overlapping fragments were amplified from cDNA. This cDNA was prepared from eluted nucleic acid from the LP of a LFD containing a clinical epithelial sample from the 2007 FMD outbreak in the United Kingdom (UKG 7B/2007) after one month's storage at 37°C. The method used was as previously described by Cottam et al, [15], with the exception that the backwards primer used for reverse transcription was UKFMD Rev (5′-GGCGGCCGCTTTTTTTTTTTTTTT-3′), which targets the poly(A) fragment of the virus genome. A 24^th^ reaction using the last forward primer and the reverse primer RACE-T21G (5′-CAGGAAACAGCTATGACTTTTTTTTTTTTTTTTTTTTTG-3′) was performed to fully obtain the poly A tract. For each PCR reaction, negative controls were performed in parallel to detect potential cross-contamination. PCR amplification products were then analysed by electrophoresis on a 1.8% agarose gel.

### Recovery of infectious FMDV from the LFDs

*In vitro* electroporation of BHK-21 (Baby Hamster Kidney) cells was used to determine whether infectious FMDV could be directly recovered from the LFDs. This involved electroporating elution wash recovered from six individual LFD sections (Figure 1) loaded with either 200 µl of O_1_ BFS Field strain or 200 µl O_1_ BFS cell culture adapted virus and an LFD to which synthetic FMDV RNA had been added were placed in 100 µl elution buffer, incubated at RT for 10 minutes prior to overlaying directly onto bovine thyroid primary cell cultures (BTY) grown in tubes. Post electroporation, two passages, each of two days duration, at 37°C on BTY cells were undertaken to observe development of cytopathic effect (CPE).

In subsequent experiments, LFDs loaded with original epithelium suspensions prepared from TUR 4/2013, TUR 2/2014 and PAK 9/2013 isolates, duplicate LFDs were prepared. One LFD from each duplicate was processed as describe above with the exception that the LP and WS were combined as one sample (LP+WS) as was the NB, NaB and NA (referred to as NC) and the elution buffer collected was frozen at −80°C for one week prior to electroporation. The second LFD from each virus was left at RT for one week prior to elution as described above before being frozen at −80°C and then electroporated.

BHK-21 cells (800 µl of 2×10^6^ cells/ml) were suspended in electroporation buffer (21 M HEPES pH 7.0, 137 mM NaCl, 5 mM KCl, 0.7 mM Na_2_HPO_4_, 6 mM glucose, made up with RNAse free water and filter sterilised) and mixed with approximately 50 µl of eluted RNA in a cuvette (0.4 cm, Bio-Rad) and subjected to a double, square wave, electrical pulse (0.5 ms) of 0.75 kV using a BioRad Gene Pulser X cell electroporation system. The electroporated cells were subsequently transferred into a 25 cm^2^ culture flask with addition of 4 mls of maintenance media (5% adult bovine serum in Glasgow eagles) and incubated at 37°C in a CO_2_ incubator for 24 hours. After freeze/thawing, supernatant was clarified via centrifugation at 13000*xg* (Hettich Rotanta 460R) and 1 ml was transferred onto a monolayer of primary BTY cells maintained in 2% adult bovine serum in Glasgow Eagles within tubes (5.5 cm^2^ Nunclone Delta tubes flat bottom) and incubated at 37°C in a CO_2_ incubator for two days prior to harvesting the clarified supernatant. Two passages, each of two days duration, at 37°C on BTY cells were undertaken to observe development of CPE. Specificity of CPE was confirmed by testing the cell culture supernatant using an antigen capture ELISA [16]–[18]. In the case of LFDs loaded with TUR 4/2013, TUR 2/2014 and PAK 9/2013 a 3D rRT-PCR was performed on the eluted FMDV as described above.

### Statistical Analysis

Differences in cycle threshold (Ct) values between LFD sections and effect of storage conditions were tested using a one-way analysis of variance (ANOVA), and where post-hoc testing was computed using the Tukey's HSD test variables associated were tested using the Analysis of Variance (ANOVA). Statistical analyses were performed and linear regression methods in R 3.1.0 [19].

## Results

### Detection of FMDV genome from RNA recovered from LFDs compared to robotically extracted RNA

**Figure 2:**
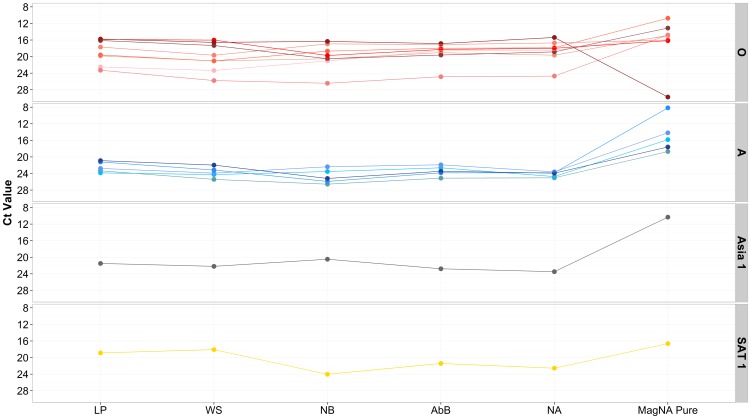
Ct values generated from FMDV 3D rRT-PCR performed on sections of 14 separate LFDs and corresponding values generated for MagNA pure extracted RNA from the equivalent original epithelial suspensions in parallel. LP: Loading Pad; WS: Wicking strip; NB: Nitrocellulose below Ab Band (NB); Nitrocellulose Ab Band (AbB); Nitrocellulose above Ab band (NA). LFDs spanned four serotypes O (red dots) (LFDs TUR 8/1969, BAR 2/2008, KUW 2/2008, SAU 3/2008, ZAM 5/2008, HKN 10/2005, IRN 53/2006, UKG 7B/2007, A (blue dots) (LFDs BAR 4/2009, IRN 1/2008, KEN 8/2008, TUR 20/2006, IRN 36/2007), Asia 1 (grey dots) (IRN 15/2001) and SAT 1 (yellow dots) (ZAM 5/2008).

It was possible to detect FMDV nucleic acid from all sections of FMDV positive LFDs with Ct values that were similar across the different sampled sections of the LFD (F_4,90_ = 0.96, p = 0.434) (Figure 2). When compared to MagNA pure extracted RNA, the Ct values derived from eluted RNA were significantly higher (F_5,108_ = 6.47, p = 0.000) with an average increase in the Ct values generated of 4.64±0.64 (p = 0.000). In addition, MagNA Pure extracted RNA had greater dynamic range in the Ct values than RNA eluted from the LFDs (95PI 16.07±0.56 to 25.27±1.01) (Figure 2).

### Effect of storage conditions upon the recovery of FMDV-specific RNA from LFDs

**Figure 3:**
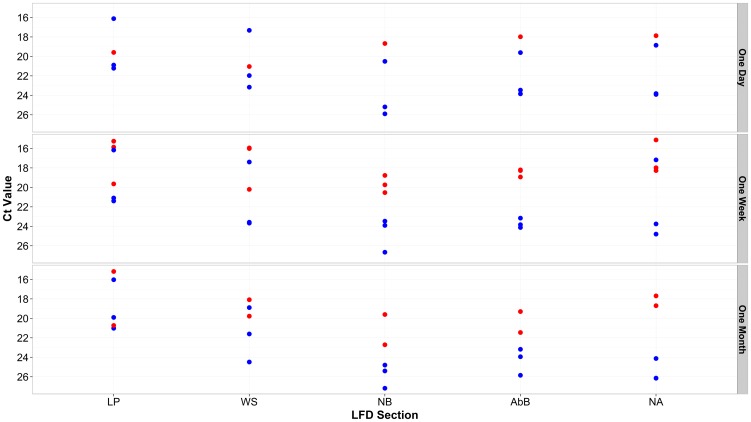
Ct values generated from FMDV 3D rRT-PCR performed on sections of 5 separate LFDs and MagNA pure extracted RNA from the original epithelial suspensions and recorded by time of LFD testing. LP: Loading Pad; WS: Wicking strip; NB: Nitrocellulose below Ab Band (NB); Nitrocellulose Ab Band (AbB); Nitrocellulose above Ab band (NA). LFDs spanned two serotypes O (red dots) (LFDs HKN 10/2005, UKG 7B/2007) and A (blue dots) (LFDs TUR 20/2006, IRN53/2006, IRN 36/2007).

FMD viral genome was detected by 3D rRT-PCR on all sections of the LFD washed with elution buffer one week and one month after visual development (Figure 3). However, the length of time that the LFD had been previous stored was not statistically associated with variation in Ct values obtained from each of the LFD section (F_2,72_ = 1.19, p = 0.311). Statistically different Ct responses were recorded for each of the LFD section (F_4,70_ = 4.16, p = 0.004) with Ct values obtained from the LP being the lowest (i.e. strongest signal), reporting an average increase of 2.75±1.09 in the Ct values produced by the other section of the LFD (p = 0.627, 0.002, 0.057, 0.236 for WS, NB, AbB and NA, respectively).

**Figure 4:**
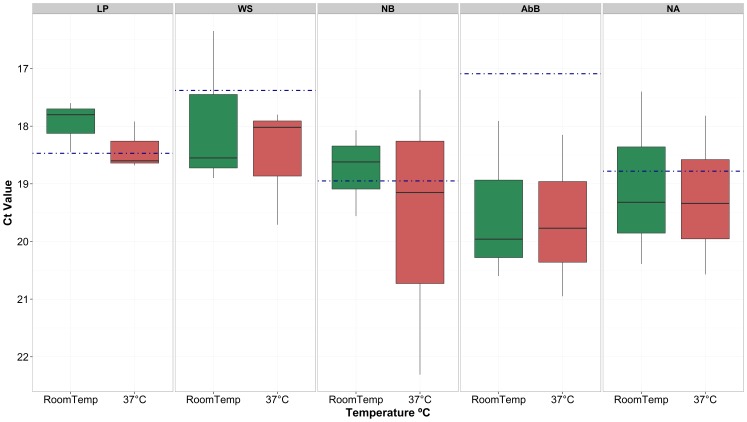
Box plot of Ct values generated from FMDV 3D rRT-PCR performed on sections of LFDs from serotype SAT 1 isolate (ZAM 5/2008) eluted at one month after LFD testing. LFDs had either been stored at room temperature (RoomT) (green bar) or 37°C (red bar). LP: Loading Pad; WS: Wicking strip; Nitrocellulose Ab Band (AbB). Dotted blue line represents baseline data (Ct values from LFDs washed on day one).

It was also possible to detect FMD viral genome by 3D rRT-PCR on all sections of the LFD (LFD loaded with ZAM 5/2008; one LFD used for baseline; triplicate LFDs used either at RT or 37°C) washed with elution buffer after one month from sample application when stored at both RT (RT, ∼25°C) and 37°C (Figure 4). Ct values derived from FMDV eluted 60 minutes post development were not statistically different to those obtained (in triplicate) from LFDs stored at RT and 37°C after one month (F_1,28_ = 0.90, p = 0.352) (95PI 17.47±0.68 to 19.58±0.93 and 17.81±0.28 to 20.48±1.30 for RT and 37°C, respectively).

### Effect of dilution on detection of FMDV genome from RNA recovered from LFDs

**Figure 5:**
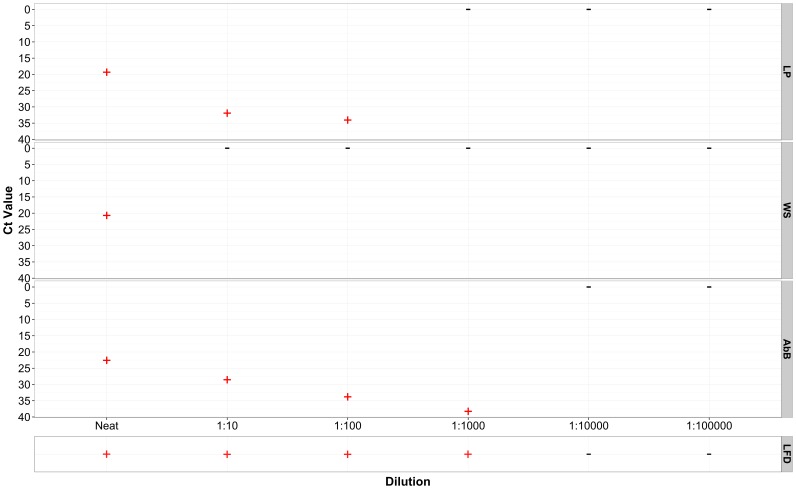
Ct values generated from FMDV 3D rRT-PCR performed on sections of LFDs in dilution series from serotype O isolate (BAR 2/2008) compared against the visual detection on the Ag LFD. LP: Loading Pad; WS: Wicking strip; Nitrocellulose Ab Band (AbB)(+)  =  genome detected; (-)  =  No Ct.

The dilution to which viral RNA could be detected was dependent upon the section of LFDs from which the RNA was eluted (Figure 5). For example it was only possible to detect viral RNA on the WS when the virus was added neat to the LFD, whereas it was possible to detect viral RNA at dilutions of 1∶1000 in elution wash derived from the AbB. The ability to detect viral genome also correlated with the ability to see the positive band on the LFD and RNA could not be detected when the antigen test-line was not present on the LFD.

### RT-PCR amplification and sequencing of VP1 from LFDs

Using a one-step RT-PCR it was possible to recover VP1 from all five sections of the LFD from serotype O isolate BAR 2/2008 (data not shown). Following gel extraction it was also possible to amplify a 1165 bp (0-1244F/EUR2B-52R) or 1135 bp (0-1272/EUR2B-52R) product containing the sequence of VP1 which matched sequence data generated from the same virus passaged once on BTY (data not shown).

### Use of RT-PCR to amplify fragments comprising the complete FMDV genome

**Figure 6:**
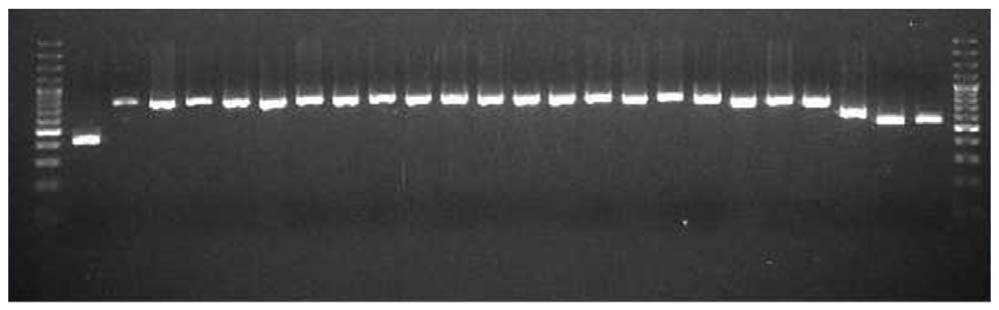
Visualization of the 24 overlapping PCR products representing the complete FMDV genome generated from the loading pad (LP) from LFD loaded with UKG 7B/2007. 1.8% agarose gel stained with ethidium bromide (0.2 µg per ml). Lane 1, S-fragment of the 5′UTR, Lanes 2–22 L fragment, Lanes 23 and 24, poly A fragment.

Using the method described by Cottam et al [15], it was possible to amplify the complete genome from RNA eluted from the LP of LFD loaded with a clinical epithelial sample (UKG 7B/2007) and which had been stored at 37°C for one month, including the S-fragment of the 5′UTR fragment of the virus (Figure 6). Since the primer used for reverse transcription targeted the poly(A)- tail at the 3′end of the FMDV genome these results provided evidence that full length FMD viral genomic RNA was present in the LFD device as a single molecule and thus electroporation to recover the virus might be achievable.

### Recovery of FMDV virus from LFDs

Attempts to recover infectious FMDV from the LP, WS and NC from LFDs loaded with O_1_ BFS field strain and O_1_ BFS cell culture adapted virus was unsuccessful when the elution wash was overlaid directly onto a monolayer of BTY cells and passaged twice on BTY cells. However when the elution wash was electroporated into BHK-21 cells and subsequently passaged onto BTY cells, infectious virus was successfully recovered correctly typed by subsequent antigen ELISA (Table 2). Similar results (with the exception of the WS from which live virus was not recovered) were obtained for the LFD containing the positive control synthetic RNA (pT7S3).

**Table 2:** Antigen (Ag) ELISA results from electroporated BHK cells passed once and twice on BTY cells.

		1^st^ BTY Passage		2^nd^ BTY passage		Ct values
Sample	LFD section	CPE	Ag ELISA	CPE	Ag ELISA	
O_1_BFS field strain	LP	+	O	+	O	Not done
	NC	+/-	-	+	O	
	WS	+/-	-	+	O	
O_1_BFS cell culture	LP	+	O	+	O	
	NC	+/-	-	+	O	
	WS	+	O	+	O	
Positive control (pT7S3)	LP	+/-	-	+	O	
	NC	+/-	-	+	O	
	WS	-	-	+/-	-	
TUR 4/2013	NC (1)	+	Not done	+	A	29.85
	LP/WS (1)	+		+	A	30.13
	NC (2)	+		+	A	33.33
	LP/WS (2)	-		-	-	28.08
TUR 2/2014	NC (1)	-		-	-	22.84
	LP/WS (1)	-		-	-	22.25
	NC (2)	-		-	-	23.75
	LP/WS (2)	-		-	-	25.11
PAK 9/2013	NC (1)	+		+	Asia 1	22.12
	LP/WS (1)	+		+	Asia 1	30.28
	NC (2)	-		-	Asia 1	22.56
	LP/WS (2)	+		+	Asia 1	23.92

LP: Loading Pad; WS: Wicking strip; NC: Nitrocellulose. +: Obvious CPE; +/-: Suspected CPE: -: No CPE. O  =  O serotype detected, A  =  A serotype detected, Asia 1  =  Asia 1 serotype detected. LP/WS: Loading Pad combined with Wicking Strip; NC: Nitrocellulose. (1)  =  LFDs washed the same day. (2)  =  LFDs washed one week later.

When we extended the investigation to an additional two serotypes (A and Asia 1) it was possible to recover infectious FMDV from both the LP/WS and NC when the elution wash was electroporated into BHK-21 cells the same day and subsequently passaged onto BTY cells from LFDs loaded with TUR 4/2013 and PAK 9/2013 (Table 2). However, following storage at RT for one week, infectious virus was only recovered from the LP (LFD loaded with PAK 9/2013), and NC (LFD loaded with TUR 4/2013). Where CPE was visible, the supernatants were positively typed by antigen ELISA to the correct serotype (Table 2). In the case of the LFD loaded with TUR 2/2014, no infectious virus was recovered; either the same day, or after one week's storage at RT. rRT-PCR was able to detect FMDV genome on all sections of the LFDs immediately following elution and also following one week's storage at RT, even in the case of LFD loaded with TUR 2/2014 which was negative for recovery of infectious FMDV.

## Discussion

This study, demonstrates for the first time, that it is possible to recover full length FMDV RNA from positive LFDs which can be successfully used as a template for diagnostic rRT-PCR, full genome amplification, sequencing and recovery of infectious virus upon electroporation. Thus this paper describes a novel methodology which could be applied for the dry, non-hazardous transportation of samples from FMD endemic countries to international reference laboratories for viral characterisation without the worry of degradation of sample. However, the analytical sensitivity of the FMDV LFD is reported to be equivalent to the antigen-detection ELISA [20] which is lower than sensitive molecular detection methods that are widely used for routine diagnostics. Therefore, this transport method is not intended to replace routine diagnostic submission for outbreak surveillance, since a negative LFD does not necessarily define a negative sample.

Regardless of the storage time or temperature, it was possible to detect FMDV genome using rRT-PCR from all sections of the LFDs for all isolates examined. These results are consistent with findings from Bisht et al, [7] and Hofmann et al, [5] who were also able to detect FMDV genome from clinical samples which had been stored for one month at elevated temperatures. However, for temperature and time of storage, the LP had significantly lower rRT-PCR Ct values and thus may be more favourable sections to use for laboratory analysis. This observation is supported by testing serial dilutions of virus applied to the LP, WS and AbB, whereby FMDV genome was still detectable at a higher dilutions for the LP and WS than other sections of the LFD.

It was also possible to amplify and sequence VP1 from all sections of the LFDs after one month of storage at RT, and amplify the complete genome from the LP after one month of storage at 37°C. In fact there was no significant difference between Ct values derived from LFD membranes stored at RT when compared to those stored at 37°C. This indicates that the LFD membranes are suitable surfaces to preserve full length RNA for extended periods of time regardless of storage temperatures. It would be of interest to determine whether these observations can be replicated for samples processed and shipped from the field and include a range of different serotypes.

A particular focus of this study was to examine whether full length FMDV RNA could be recovered from the lateral flow membranes. Following electroporation, it was possible to recover infectious FMDV that was correctly typed by antigen ELISA. Although LFDs have not been previously tested, these findings are consistent with published work [6] that describes recovery of infectious FMDV from clinical samples preserved in RNA storage buffers. Electroporation of elution wash one week following development on the device was less successful for the recovery of infectious virus. This observation is consistent with data reported by Hofmann et al, [5], whereby the ability to recover infectious virus following electroporation of RNA stored in Trizol declined over time. The success of recovering virus was not related to the section used on the lateral flow device and suggests that should this method be adopted for recovery of virus it would be important to electroporate elution washes from multiple LFD sections to optimise recovery of infectious full length RNA. Future studies should extend the storage time and include elevated temperatures to determine the point at which infectious virus can no longer be recovered.

Contrary to the findings by Belsham et al, [6] the rRT-PCR Ct values in this study did not appear to correlate with the ability to recover infectious virus. For example we were able to recover infectious virus from TUR 4/2013 (serotype A) and PAK 9/2013 (serotype Asia 1) which had average Ct values of 30±2 and 25±4 respectively, yet we were unable to recover infectious virus from TUR 2/2014 (serotype Asia 1) despite average Ct values of 23±1. The findings of this study are therefore more consistent with those published by Bisht et al, [7] who also reported varied success in recovery of infectious virus from RNA extracted from clinical samples despite strong multiplex PCR results [7]. Further evaluation is required on a greater number of isolates to determine the optimum method/section of LFD to use for recovery of infectious FMDV RNA.

Infectious virus was not recovered (absence of CPE) following direct passage of the elution wash onto BTY cells. It is already known that purified RNA (non-encapsulated) is not infectious when inoculated onto susceptible cells for CSFV [21] and therefore our results suggest that when FMDV is applied to the lateral flow device, the viral capsid does not remain intact but disassociates releasing the RNA for preservation on the membrane. This is a significant finding as it suggests that positive lateral flow devices may pose little to zero-biorisk should they be used for transportation of samples between the field and reference laboratories. In view these data, further work to consider and agree biosecurity guidelines is required so that these new methods can be transitioned into the field for the safe preservation and recovery of FMDV. Furthermore, we aim to continue this work using additional clinical samples added to the LFD in the field which have been shipped back to the WRLFMD and to compare directly to routine virus isolation methods in order to determine whether there is a difference in the efficiency of infectious virus recovery.
